# Simulation study of frailty as a baseline confounder: the need to improve reporting intensive care trials

**DOI:** 10.1186/s13054-026-06118-2

**Published:** 2026-06-02

**Authors:** Christopher Dugan, Suzanne Weightman, Anders Aneman

**Affiliations:** 1 Intensive Care Unit, Clinical Building 2, Liverpool Hospital, South Western Sydney Local Health District, Locked Bag 7103, Liverpool, 1871 NSW BC Australia; 2 University of New South Wales, Sydney, Australia

**Keywords:** Frailty, Confounding variable, Critical care

## Abstract

**Background:**

Frailty is an independent predictor of worse outcomes after critical illness, yet intensive care trials almost invariably omit reporting frailty distribution in the investigated cohort. This study investigated the magnitude and prevalence of imbalanced baseline distribution in frailty using the ordinal Clinical Frailty Scale that could contribute to clinically significant differences in apparent mortality.

**Methods:**

A database of 6968 patients admitted to intensive care unit was used to simulate enrolment of between 100 and up to 1000 patients into each of the control and interventional arms of a hypothetical two-arm trial. Both 1:1 randomisation and block randomisation using permuted blocks of variable sizes were simulated 100 times and referred to as ‘trials’. The observed relationship between the Clinical Frailty Scale and observed in-hospital mortality was used to determine thresholds for frailty imbalance sufficient to generate ≥2% and ≥5% differences in mortality. The proportions of ‘trials’ meeting these criteria are reported as percentages and 95%CI.

**Results:**

Imbalanced frailty generated a ≥2% mortality difference in 36 [95%CI 31-42]% of ‘trials’ with 100 patients/arm that was reduced to 9 [95%CI 6-13]% of ‘trials’ with 400 patients/arm. An imbalance in frailty associated with a ≥5% mortality difference occurred in 3 [95%CI 1-5]% of ‘trials’ with 100 patients/arm and reduced to 0% [95%CI 0-1.3]% in all ‘trials’ using larger sample sizes. ‘Trials’ with a binary balance between non-frail vs frail patients still retained an ordinal frailty imbalance to generate a ≥2% mortality difference in 10 [95%CI 7-14]% with 100 patients/arm. Block randomisation only reduced the proportion of ‘trials’ with mortality differences related to frailty to a minor and variable degree.

**Conclusions:**

Baseline distributional imbalance in frailty may create clinically significant differences in apparent mortality estimates. The hypothesis-generating findings in this study suggest that intensive care trials should report complete frailty distributions and consider frailty in study designs and analyses.

**Supplementary Information:**

The online version contains supplementary material available at 10.1186/s13054-026-06118-2.

## Background

Frailty is a multi-system syndrome with large inter-individual variation that associates with worse outcomes after critical illness [1, 2]. While partially correlated with age and comorbidities, it remains an independent prognostic factor [3, 4]. The Acute Physiology and Chronic Health Evaluation score (APACHE) is a summary metric of age, comorbid state and acute physiological variables that predicts in-hospital mortality [5] and typically used to describe baseline patient acuity in intensive care trials. The Clinical Frailty Scale (CFS) [6] is the most validated tool to assess frailty in critically ill patients, based on the health status in the two weeks preceding hospital admission.

Baseline frailty can be a confounder in ICU trials if associated with treatment group selection and patient outcome or prognostic imbalance and an effect modifier if it impacts the magnitude or direction of the treatment response. It should therefore arguably be reported [7] and, if appropriate, adjusted for in statistical analyses of intensive care trials [8]. Despite the prognostic importance of frailty, intensive care trials almost invariably omit reporting frailty distribution in the investigated cohort. The central tendency and high internal validity of well conducted large trials may overcome this omission, but residual confounding by imbalanced frailty cannot be excluded. The average effect size reported in a randomised clinical trial may apply differently to patients with disparate frailty. Subgroup analyses of frailty are typically underpowered and interaction terms often omitted. This study used patient data from an ICU admissions database to simulate recruitment into a trial with the observed in-hospital mortality set as the outcome. Three aims were addressed in the simulations using real life data. First, to determine what magnitude of distributional imbalance in frailty that creates clinically meaningful differences in apparent mortality between trial arms. Second, to describe the frequency of such distributional frailty imbalance across trial sample sizes representative of ICU studies. Third, to examine the proportion of trials in which an imbalanced ordinal distribution of frailty persists when the general dichotomous distribution of non-frail vs frail patients is well balanced.

## Methods

The dataset used in this study comprised routine reports from Liverpool Hospital ICU to the Australian New Zealand Intensive Care Society (ANZICS) Adult Patient Database. The reports are prospectively generated by a professional data manager including rigorous quality checks. This database contains all variables for APACHE III as well as ICU and hospital outcomes. The study was approved by the Human Ethics Research Committee, South Western Sydney Local Health District (2022/ETH01662) with waiver of participants’ informed consent. Liverpool ICU is a tertiary academic unit affiliated with the University of New South Wales with approximately 3000 admissions per annum and is a major contributing site to ANZICS clinical trials. The CFS of all patients admitted to the ICU has been recorded since May 2018 [9–11] by the treating intensivist or senior intensive care trainee based on review of the patient, the electronic medical record and discussions with next of kin. The standardisation of CFS assessments is maintained by the contemporaneous recording at the time of admission by a limited group of trained, experienced clinicians using instructions and visual aids approved by the ANZICS data committee. The accuracy of these routinely captured data is accepted for ANZICS performance and benchmarking processes and no further quality control or reliability measures were conducted specific to this study. The CFS assesses frailty according to an eight point ordinal scale: 1, very fit; 2, well; 3, managing well; 4, vulnerable; 5, mildly frail; 6, moderately frail; 7, severely frail; 8, very severely frail (an additional Category 9 for terminally ill patients with a life expectancy < 6 months was not included in line with the ANZICS user guideline, [12]). A dichotomous definition of non-frail (CFS 1-4) vs frail (CFS ≥5) patients [3, 6, 13, 14] was also applied. The source population was all patients admitted between May 2018 and September 2024. The dataset including CFS was further restricted to non-pregnant patients admitted to ICU for full active support without any limitations to treatment, as these criteria are commonplace to assess eligibility for clinical trials. 

**Fig. 1:**
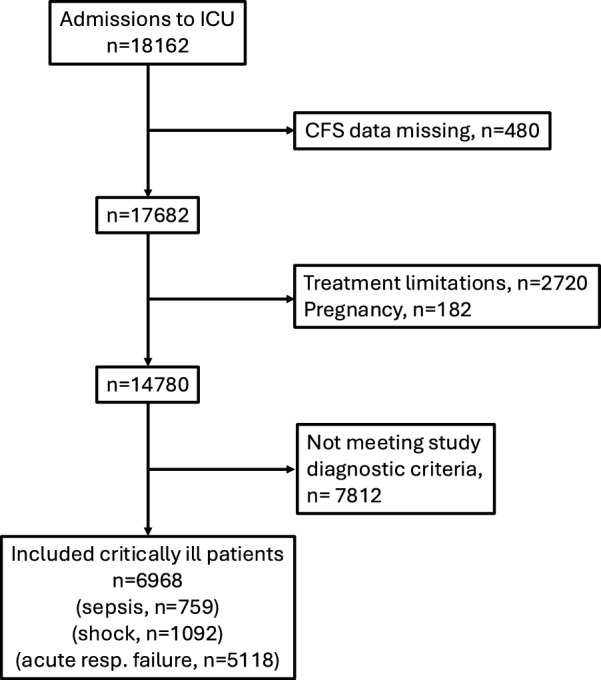
Flow chart of the study source population

Three diagnostic cohorts were identified from the dataset using APACHE III physiological variables and diagnostic codes. These cohorts generally represent critically ill patients and specifically constitute typical target populations in ICU trials: 1) shock defined as either a systolic blood pressure < 90 mmHg or a mean arterial pressure < 60 mmHg and an arterial lactate level > 2 mmol^.^l^-1^; 2) sepsis defined by a final diagnostic code of sepsis and an arterial lactate level > 2 mmol^.^l^-1^; and 3) acute respiratory failure defined as receiving either invasive or non-invasive mechanical ventilatory support and with a partial oxygen pressure (mmHg) to inspired fraction of oxygen ratio of < 300 mmHg (Fig. 1).

### Trial simulation procedure

The rationale underpinning this study is the random selection of subjects from a large pool of patients into the control and interventional arms of a hypothetical two-arm, parallel group randomised clinical trial. Random selection was performed in pairs to represent 1:1 randomisation, as well as by block randomisation using permuted blocks of variable sizes (4, 6 and 8 patients). This procedure was used to replicate recruitment of patients with their inherent variability carried forward into a randomised controlled trial of a notional intervention. The observed in-hospital mortality was set as the primary outcome. Sample sizes ranged from 100 to 1000 patients per arm to encompass pilot or proof of concept trials (100 patients/arm), typical ICU trials (400-500 patients/arm), and large multicentre trails (750-1000 patients/arm), consistent with published reviews [15, 16]. Sampling with replacement preserved distributional properties of the cohort if the targeted sample size exceeded the available cohort size. The random selection was repeated to produce 100 replicates that are referred to as ‘trials’ and performed for all sample sizes and all types of randomisations. 

### Balance assessment

Standardised mean differences were calculated to assess the balance of distributions in age, gender, APACHE III, CFS strata 1-8 and the CFS dichotomised to non-frail vs frail patients. The two arms of a ‘trial’ were considered balanced if the standardised mean difference (SMD) was < 0.1 [17]. The mean distribution of CFS in each ‘trial’ arm and the absolute mean CFS difference were also calculated. The mean CFS difference represents the weighted sum of ordinal shifts and provides an aggregate metric of CFS distribution [2] in a single, interpretable threshold.

The three diagnostic cohorts were treated as populations in which frailty (CFS ≥5) was considered an exposure to calculate the population attributable fraction (see Additional file 1 for details of all calculations).

### Distributional imbalance in frailty and mortality differences

Objective, data-driven thresholds in CFS distributions were calculated, using both the dichotomous non-frail vs frail and the ordinal CFS, that would generate ≥2% and ≥5% differences in hospital mortality between the two ‘trial’ arms. The percentages were set *a priori* to represent differences likely to significantly change the interpretation of intensive care trials. The 2% difference could change the certainty in a mortality point estimate and represents the typical observed effect size in multicentre clinical trials [15, 16]. A 5% difference could represent a minimal clinically important difference [18, 19]. The thresholds were calculated using the observed CFS-mortality relationship in each cohort. Shifts across thresholds were calculated as the percentages of the population that must move on the ordinal CFS to create mortality differences of ≥2% and ≥5% as well as the corresponding changes in the absolute mean CFS. All thresholds were identified from the smallest shifts of any direction sufficient to generate the mortality differences. This bidirectional approach ensured distributional imbalance was detected regardless of with ‘trial’ arm had the higher mean frailty, as either direction creates equivalent confounding.

### Persistent ordinal imbalance in studies balanced for non-frail vs. frail patients

‘Trials’ achieving a dichotomous balance (SMD <0.1 for non-frail vs frail proportions) but retaining ordinal CFS distributional imbalance sufficient to generate ≥2% mortality difference are termed ‘paradoxical trials’. This terminology reflects the paradox that such ‘trials’ simultaneously satisfy conventional balance criteria (binary SMD <0.1) while exhibiting clinically meaningful prognostic imbalance. These ‘trials’ represent hidden confounding that would be missed by standard reporting practices.

### Statistical analyses

Descriptive statistics were used to report the study dataset. The correlations between CFS and age and the APACHE III score were assessed by Spearman’s rho. For each sample size and diagnostic cohort, the proportion of ‘trials’ with distributional imbalance was calculated from 100 simulated ‘trials’. A number of 100 ‘trials’ provided a maximum ±5% margin of error at the 95% confidence level across the observed range of prevalence for imbalanced frailty at 8-38% in pilot runs, and hence 100 simulations was accepted to provide sufficient precision in the prevalence estimates. Point estimates (proportions as percentages) are shown as dots, with Wilson score 95% confidence intervals shown as error bars to illustrate the stability of estimates. Trend lines were fitted using locally estimated scatterplot smoothing (LOESS, span = 0.75) for visualization. All analyses were performed in RStudio (Integrated Development Environment for R, version 2023.06.0, Posit Software, PBC, Boston MA, USA). The R scripts were written with assistance from Claude (Sonnet 4.5, Anthropic 2025, https://www.anthropic.com). All scripts and outputs were reviewed and verified by the authors. Annotated sections of the R code used for threshold calculations, assessment of paradoxical ‘trials’ and simulations with permuted block randomisation are provided in the Additional file 1.

## Results

The dataset comprised 6968 eligible patients from 18162 total admissions. The characteristics of each cohort are reported in Table 1. A diagnostic overlap with sepsis was present in 286 patients with shock and 1080 patients with acute respiratory failure.

**Table 1:** Patient characteristics and clinical frailty scale (CFS) distribution for the dataset from which ‘trials’ were sampled

Variable	Critically ill	Shock	Sepsis	Acute resp. failure
	n=6968	n=1091	n=759	n=5118
**Demographics**				
Age, years, median (IQR)	63.8 (52.1–73.2)	64.4 (50.3–73.4)	64.5 (52.2–73.8)	63.7 (52.6–73.1)
Male gender, n (%)	4214 (60)	660 (61)	479 (63)	3366 (66)
**Clinical characteristics**				
APACHE III, median (IQR)	56.0 (43.0–75.0)	63.0 (47.0–83.0)	70.0 (54.0–89.0)	55.0 (42.0–73.0)
ICU LOS, days, median (IQR)	3.1 (1.8–6.1)	3.2 (1.8–6.6)	3.5 (1.8–7.7)	3.2 (1.9–6.2)
ICU mortality, n (%)	443 (6.4)	128 (12)	83 (11)	305 (6)
Hospital LOS, days, median (IQR)	13.1 (7.1–25.2)	12.0 (6.3–23.4)	16.0 (8.1–36.0)	13.2 (7.3–25.2)
Hospital mortality, n (%)	725 (10)	168 (15)	140 (18)	527 (10)
**Clinical frailty scale, n (%)**				
CFS 1	233 (3.3)	36 (3.3)	17 (2.2)	180 (3.5)
CFS 2	1047 (15)	192 (18)	72 (9.5)	783 (15)
CFS 3	1950 (28)	275 (25)	181 (24)	1494 (29)
CFS 4	1831 (26)	219 (20)	218 (29)	1394 (27)
CFS 5	797 (11)	127 (12)	94 (12)	576 (11)
CFS 6	813 (12)	181 (17)	109 (14)	523 (10)
CFS 7	260 (3.7)	52 (4.8)	62 (8.2)	146 (2.9)
CFS 8	37 (0.5)	9 (0.8)	6 (0.8)	22 (0.4)

**Fig. 2:**
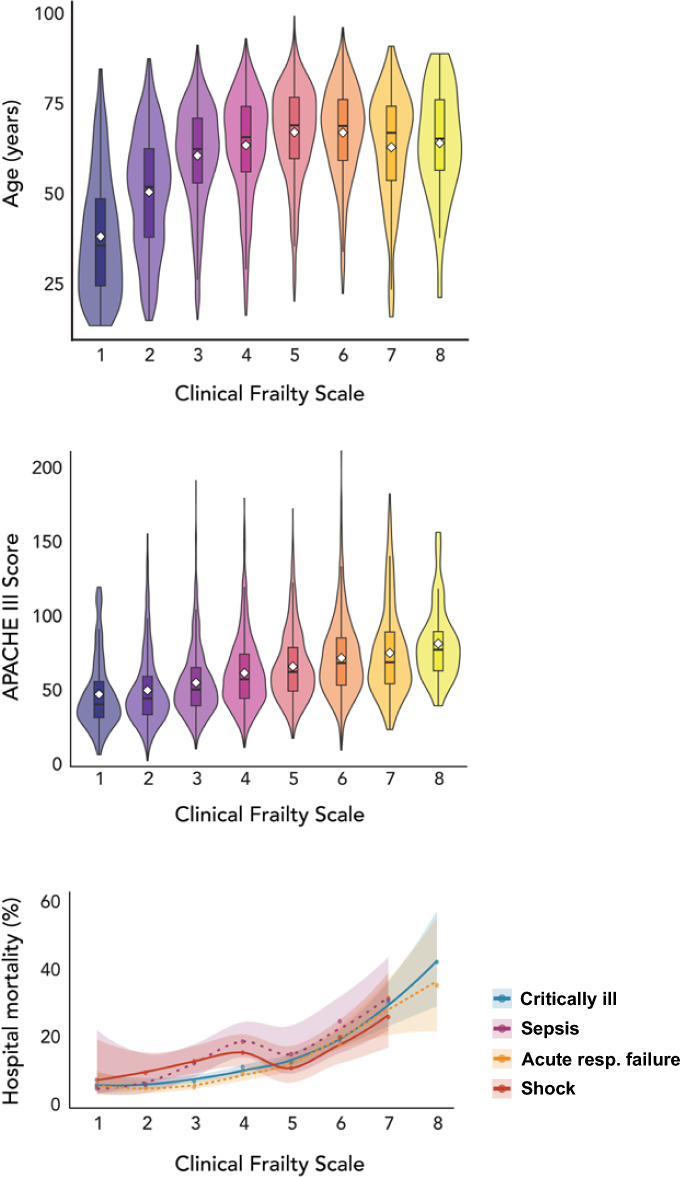
Violin plots showing the density shape for age (top) and APACHEIII (middle) distributions across the Clinical Frailty Scale (CFS) in the overall cohort of critically ill patients (n=6968). The full distribution is shown with the box and line denoting the interquartile range and median, and the mean denoting the diamond. The in-hospital mortality across the CFS (bottom) is shown for the cohort of critically ill (solid magenta line) as well as the diagnostic subgroups (sepsis dashed purple line; acute respiratory failure; dotted orange line; shock solid red line) with the shaded areas indicating the 95% confidence intervals

Figure 2 shows age, APACHE III, and mortality distributions across CFS. The correlation between CFS and age was rho=0.34 [0.32-0.37], p<0.001, and for APACHE III rho=0.33 [0.31-0.35], p<0.001. Hospital mortality increased from CFS 1 to 4 but most markedly at CFS ≥ 5. The prevalence of CFS 8 was <1% in all cohorts and was not explored further. Mortality attributable to frailty was 13% [2.7-23] in shock, 19% [7.5-30] in sepsis and 26% [20–31] in acute respiratory failure.

**Fig. 3:**
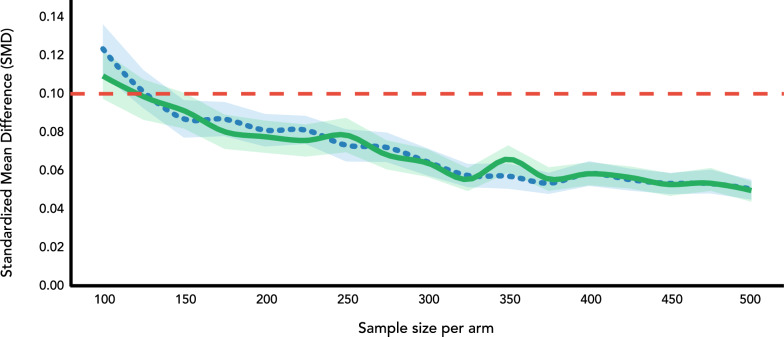
Standardised mean difference (SMD) for age (blue dotted line) and APACHEIII (green solid line) observed in ‘trials’ of increasing sample size. The shaded areas indicate the 95% confidence intervals. The dashed red line indicate the SMD of 0.1 typically accepted to reflect a well-balanced distribution [17]

The SMD both for age and APACHE III SMD in ‘trials’ with 100 patients/arm was 0.12, falling below <0.1 beginning at ≥ 125 patients/arm (Fig. 3).

**Fig. 4:**
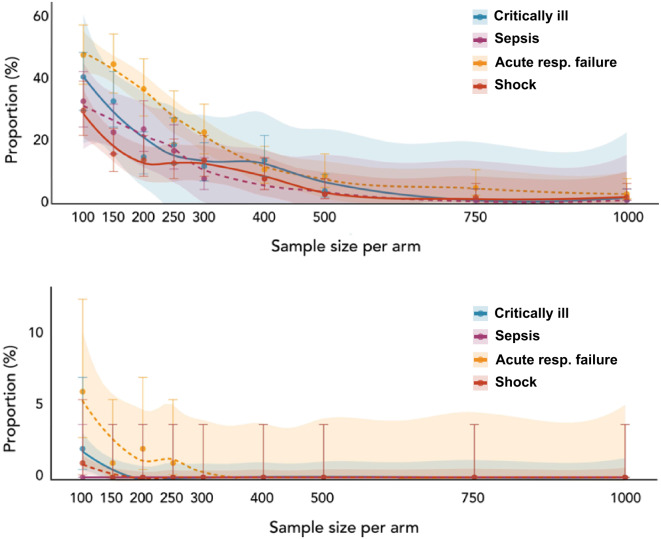
Prevalence of ‘trials’ of increasing sample size for which the distributional imbalance in the Clinical Frailty Scale (CFS) was sufficient to generate a difference of ≥2% (top) and ≥5% (bottom) in hospital mortality shown for critically ill (solid magenta line), sepsis (dashed purple line), acute respiratory failure (dotted orange line) and shock (solid red line). Graphs show point estimates with the Wilson confidence intervals indicated by error bars with LOESS smoothed trend lines

The ≥2% and ≥5% differences in hospital mortality between the two ‘trial’ arms were achieved in all simulations (data not shown). The bidirectional thresholds for distributional imbalance are reported in Table 2 with details in Additional file 1, Table S1. Mean CFS differences in the minimum to maximum range of of 0.14-1.55 (depending on diagnostic cohort and shift type) were sufficient to create ≥2% mortality difference. For the 5% threshold, mean CFS differences of in the minimum to maximum range of 0.36-2.87 were required (for other thresholds see Additional file 1, Table S1). Upward shifts (less frail → more frail) and downward shifts (more frail → less frail) demonstrated symmetrical thresholds, hence only the absolute degree of distributional imbalance mattered. Within the frail category (CFS 5-7) even single-step ordinal shifts of 8-15% of patients in the cohorts or a mean CFS difference of 0.21-0.60, were sufficient to generate a ≥2% mortality difference. Shifts generating a mortality difference of ≥5% showed the same pattern but with the least shift needed at 20-37% of the cohorts or a mean CFS difference of >0.51. The proportion of ‘trials’ of different sample sizes in which these CFS distributional imbalance criteria occurred are shown in Fig. 4 with details in Table 3. ‘Trials’ of acute respiratory failure needed more than 500 patients/arm to reduce the proportion of imbalanced frailty associated with ≥2% mortality difference below 10%, while this threshold was achieved with more than 300 patients/arm in critically ill, sepsis and shock patients. The proportion of imbalanced ‘trials’ associated with ≥5% mortality difference was 1-9% across all cohorts at 100 patients/arm and the mean proportion decreased to 0% in larger sample sizes.

**Table 2:** Data-driven threshold criteria for shifts on CFS needed to generate a ≥2% and ≥5% difference in in-hospital mortality

Cohort	Δ mortality	CFS shift	% shift	Mean CFS change
Critically ill	≥2%	1-4 ↔ 5	44.5	0.87
	≥2%	1-4 ↔ 6	19.4	0.57
	≥2%	1-4 ↔ 7	9.39	0.37
	≥2%	5 ↔ 6	34.1	0.34
	≥2%	5 ↔ 7	11.9	0.24
	≥2%	6 ↔ 7	18.3	0.18
Critically ill	≥5%	1-4 ↔ 5	65.3	2.17
	≥5%	1-4 ↔ 6	48.2	1.42
	≥5%	1-4 ↔ 7	23.5	0.93
	≥5%	5 ↔ 6	85.2	0.85
	≥5%	5 ↔ 7	29.6	0.59
	≥5%	6 ↔ 7	45.7	0.46
Shock	≥2%	1-4 ↔ 5	52.3	1.54
	≥2%	1-4 ↔ 6	26.5	0.81
	≥2%	1-4 ↔ 7	14.8	0.60
	≥2%	5 ↔ 6	21.8	0.22
	≥2%	5 ↔ 7	13.2	0.26
	≥2%	6 ↔ 7	33.7	0.34
Shock	≥5%	1-4 ↔ 5	76.8	2.35
	≥5%	1-4 ↔ 6	66.1	2.03
	≥5%	1-4 ↔ 7	37.1	1.51
	≥5%	5 ↔ 6	54.5	0.54
	≥5%	5 ↔ 7	33.1	0.66
	≥5%	6 ↔ 7	84.3	0.84
Sepsis	≥2%	1-4 ↔ 5	34.3	1.55
	≥2%	1-4 ↔ 6	18.6	0.52
	≥2%	1-4 ↔ 7	11.6	0.44
	≥2%	5 ↔ 6	20.6	0.21
	≥2%	5 ↔ 7	12.3	0.25
	≥2%	6 ↔ 7	30.4	0.30
Sepsis	≥5%	1-4 ↔ 5	64.9	2.87
	≥5%	1-4 ↔ 6	46.6	1.29
	≥5%	1-4 ↔ 7	28.9	1.09
	≥5%	5 ↔ 6	51.4	0.51
	≥5%	5 ↔ 7	30.7	0.61
	≥5%	6 ↔ 7	76.1	0.76
Acute resp. failure	≥2%	1-4 ↔ 5	34.0	0.66
	≥2%	1-4 ↔ 6	18.1	0.53
	≥2%	1-4 ↔ 7	8.00	0.31
	≥2%	5 ↔ 6	38.5	0.38
	≥2%	5 ↔ 7	10.5	0.21
	≥2%	6 ↔ 7	14.4	0.14
Acute resp. failure	≥5%	1-4 ↔ 5	85.0	1.64
	≥5%	1-4 ↔ 6	45.1	1.32
	≥5%	1-4 ↔ 7	20.0	0.79
	≥5%	5 ↔ 6	96.2	0.96
	≥5%	5 ↔ 7	26.2	0.52
	≥5%	6 ↔ 7	35.9	0.36

Bidirectional shifts (↔) mean that movements can occur in either direction between trial arms

**Table 3:** Prevalence of distributional imbalance sufficient to generate a ≥2% or ≥5% difference in hospital mortality in ‘trials’ of different sample sizes

Cohort	n /arm	Δ mortality ≥2%	Δ mortality ≥5%	Paradoxical ‘trials’
Critically ill	100	36.0 (30.8-41.6)	2.7 (1.4-5.2)	9.7 (6.8-13.5)
	200	17.3 (13.5-22.0)	0.0 (0.0-1.3)	3.7 (2.1-6.4)
	300	10.3 (7.4-14.3)	0.0 (0.0-1.3)	3.7 (2.1-6.4)
	400	9.0 (6.3-12.8)	0.0 (0.0-1.3)	2.7 (1.4-5.2)
	500	4.3 (2.5-7.3)	0.0 (0.0-1.3)	0.3 (0.1-1.9)
	750	0.3 (0.1-1.9)	0.0 (0.0-1.3)	0.0 (0.0-1.3)
	1000	0.0 (0.0-1.3)	0.0 (0.0-1.3)	0.0 (0.0-1.3)
Shock	100	37.3 (32.1-42.9)	3.3 (1.8-6.0)	4.0 (2.3-6.9)
	200	16.0 (12.3-20.6)	0.3 (0.1-1.9)	3.0 (1.6-5.6)
	300	8.0 (5.4-11.6)	0.0 (0.0-1.3)	1.3 (0.5-3.4)
	400	3.0 (1.6-5.6)	0.0 (0.0-1.3)	0.3 (0.1-1.9)
	500	3.0 (1.6-5.6)	0.0 (0.0-1.3)	0.0 (0.0-1.3)
	750	0.0 (0.0-1.3)	0.0 (0.0-1.3)	0.0 (0.0-1.3)
	1000	0.0 (0.0-1.3)	0.0 (0.0-1.3)	0.0 (0.0-1.3)
Sepsis	100	36.0 (30.8-41.6)	1.0 (0.3-2.9)	8.0 (5.4-11.6)
	200	17.3 (13.5-22.0)	0.0 (0.0-1.3)	3.3 (1.8-6.0)
	300	10.3 (7.4-14.3)	0.0 (0.0-1.3)	1.3 (0.5-3.4)
	400	4.3 (2.5-7.3)	0.0 (0.0-1.3)	0.3 (0.1-1.9)
	500	3.7 (2.1-6.4)	0.0 (0.0-1.3)	0.3 (0.1-1.9)
	750	0.3 (0.1-1.9)	0.0 (0.0-1.3)	0.0 (0.0-1.3)
	1000	0.3 (0.1-1.9)	0.0 (0.0-1.3)	0.0 (0.0-1.3)
Acute resp. failure	100	47.0 (41.4-52.7)	9.0 (6.3-12.8)	15.3 (11.7-19.8)
	200	32.3 (27.3-37.8)	2.0 (0.9-4.3)	8.7 (6.0-12.4)
	300	20.0 (15.9-24.9)	0.0 (0.0-1.3)	7.7 (5.2-11.2)
	400	16.3 (12.6-20.9)	0.3 (0.1-1.9)	7.0 (4.6-10.5)
	500	11.0 (7.9-15.0)	0.0 (0.0-1.3)	4.0 (2.3-6.9)
	750	2.7 (1.4-5.2)	0.0 (0.0-1.3)	1.7 (0.7-3.8)
	1000	0.3 (0.1-1.9)	0.0 (0.0-1.3)	0.0 (0.0-1.3)

Paradoxical ‘trials’ were defined as ‘trials’ with well balanced (SMD <0.1) binary distributions of non-frail vs frail but with CFS distributional imbalance still sufficient to generate a ≥2% mortality difference. Values are percentages with 95% confidence intervals in parentheses

**Fig. 5:**
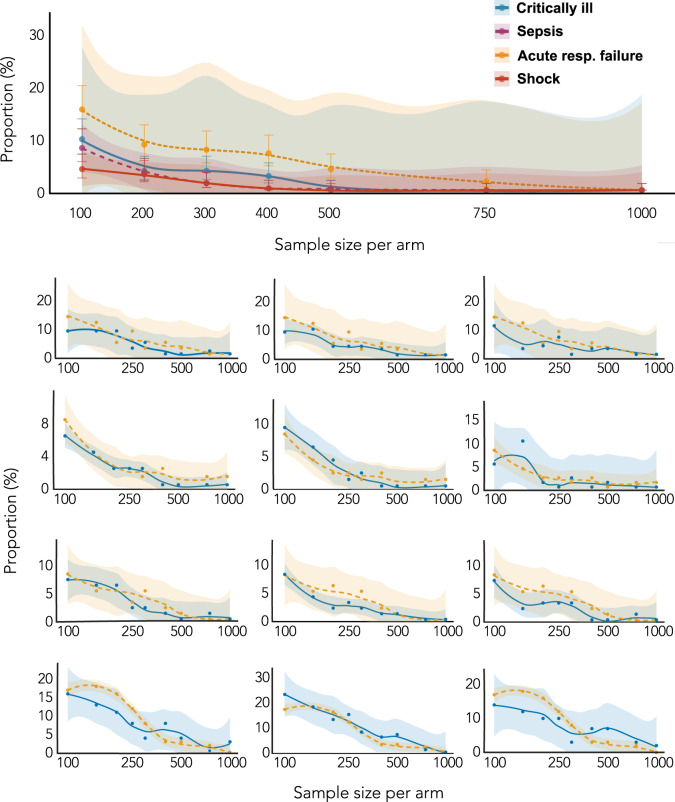
Prevalence of paradoxical ‘trials’ of increasing sample size for which the binary distributions of non-frail vs frail patients were well balanced (SMD <0.1) between the two arms using 1:1 randomisation, but the ordinal CFS distributions still imbalanced to generate a ≥2% difference in hospital mortality (top large panel) for critically ill (solid magenta line), sepsis (dashed purple line), acute respiratory failure (dotted orange line) and shock (solid red line). Prevalences using block randomisation with sizes 4-8 (blue solid lines) were compared to 1:1 randomisation (yellow dotted lines) (bottom set of panels) for critically ill (top), shock (top middle), sepsis (bottom middle) and acute respiratory failure (bottom). Graphs show point estimates with the Wilson confidence intervals indicated by error bars with LOESS smoothed trend lines

The proportion of paradoxical ‘trials’ across sample sizes is shown in Fig. 5 (top) with details in Table 3. The proportion of paradoxical ‘trials’ ranged from 4-15% with 100 patients/arm across diagnostic cohorts, corresponding to approximately 1 in 8 pilot / proof of concept trials that would pass conventional binary balance checks while retaining clinically meaningful distributional imbalance. This proportion decreased to <10% for typical ICU trials with ≥400 patients/arm. Randomisation by permuted blocks of 4, 6, and 8 patients reduced the proportion of paradoxical scenarios to a variable and minor degree that remained at 5-10% in critically ill patients overall at 250 patients/arm (Fig. 5, bottom).

## Discussion

This simulation study of 6,968 critically ill patients demonstrated that distributional frailty imbalance sufficient to generate an apparent mortality difference by ≥2% occurred in approximately one-third of 100-patient/arm trials and one in seven 400-patient/arm trials, despite well-balanced age and APACHEIII. More severe imbalance generating a ≥5% mortality difference occurred in less than one in ten ‘trials’ and limited to the smallest sample of 100-patient/arm. One in seven 100-patient/arm ‘trials’ with well-balanced binary non-frail vs frail distributions retained ordinal CFS imbalance causing ≥2% mortality difference, becoming negligible only at ≥400 patients/arm. These results highlight the need to report stratified baseline frailty distributions in ICU trials.

The demographics in healthcare settings in which most ICU trials are conducted are changing with an expanding older, more complex comorbid population admitted for advanced interventions [20]. The routinely reported age and APACHEIII achieved a SMD <0.1 already at ‘trials’ of 100-125 patients/arm, common to feasibility and pilot studies, meaning that very few ICU trials would appear poorly balanced using these baseline variables. Frailty captures the overall function of a patient that is related to but not contained by the simple presence of comorbid diagnoses as captured by APACHEIII. The substantial mortality attributable to frailty further supports its importance as a distinct baseline prognostic variable and its prognostic importance is well documented for shock [21–24], sepsis [25–28] and acute respiratory failure [29–32]. While frailty seems increasingly recognised in the design of clinical trials of pharmacological interventions [33–35], it remains absent in published statistical analysis plans for ICU trials with very few recent exceptions [36–38]. At the minimum sample size adequately balancing age and APACHEIII, 36% of ‘trials’ were sufficiently imbalanced to generate an in-hospital mortality difference of 2%. In two reviews of powering bias and clinically important treatment effects for trials in critically ill patients, the median total sample size was 843 [IQR 411-1588] and 1006 [538-2403] for 101 and 52 multicentre superiority trials with patient-level randomisation and mortality as the endpoint [15, 16]. The observed mortality differences were a median -0.2 [IQR -1.7-2.0] % and 2.0 [IQR 0.8-3.9] %. At this mortality difference and median sample size, the proportion of simulated ‘trials’ with imbalanced frailty sufficient to generate an equal difference in the mortality estimate was 13% in critically ill. The proportions within the separate diagnostic cohorts were slightly lower at 7-10% of ‘trials’. While the simulated ‘trials’ in this study cannot fully reproduce the nuances of real-life studies, the result that imbalanced frailty in one out of seven to one out of 14 ‘trials’ generated an apparent mortality difference at the typical effect estimate and sample size of actual studies, underscores the need to include frailty distribution as a baseline characteristic. Improving the precision of effect estimates in mid-sized ICU trials is important since these are influential in calculations of treatment effects and power when larger trials are designed [39]. Reporting frailty distribution could achieve a less biased, and probably in most cases reduced effect size estimate, to reduce delta inflation by overestimating the true population effect size [40, 41].

While an apparent mortality difference similar to a ‘realistic’ 2% risk difference [15, 16] cannot be ignored, the impact on greater mortality differences was much less pronounced. The proportion of ‘trials’ with a 5% mortality difference, set to equal the minimal clinically important treatment effect of a definitive randomised clinical trial what would change routine clinical practice [15, 42], was negligible in sample sizes well balanced for age and APACHEIII.

Paradoxical ‘trials’ that retained imbalanced ordinal CFS distribution while appearing well balanced by the common dichotomous definition of frailty [2, 6, 14] may contribute to treatment effect heterogeneity by residual confounding. Frailty can also contribute to heterogeneity if linked to the proportion of trial participants that are responsive to the intervention. Heterogeneity of treatment effects is increasingly recognised in ICU trials [43] and may limit achieved statistical power [16]. The use of block randomisation did not substantially reduce the proportion of paradoxical ‘trials’ that cannot be remedied by conventional regression adjustment for binary frailty if the ordinal imbalance that drives mortality differences is not recognized. Adding frailty as a covariate could increase study power without risk of overfitting statistical models by including the complete CFS distributions [44]. At a sample size of 250 patients/arm, paradoxical imbalance occurred in less than one in 20 ‘trials’ and is therefore unlikely to represent a major confounder in study cohorts typical for ICU studies [15, 16].

This simulation study is strengthened by using a large dataset of patients admitted to ICU with frailty documented by the widely used CFS and diagnostic cohorts identified using objective criteria often applied in clinical trials. The potential selection and information biases were therefore minimised. The distribution of CFS values were calibrated against actual observed in-hospital mortality to generate objective, data-driven imbalance thresholds against 2% and 5% mortality differences. Simulation of 100 ‘trials’ produced robust confidence intervals, while fewer replicates would arguably be closer to the number of trials available to inform clinical recommendations. The dispersion of distributional imbalance of frailty can be expected to increase with fewer ‘trials’, with the impact on effect estimates ranging from negligible to substantial. The study is limited by using data from a single ICU and the observed hospital mortality may not be representative of other institutions. Furthermore, mortality censored later in the clinical trajectory for example at 90 days [45] may better capture the sustained impact of frailty, but these data were not available in this study. In cohorts with higher mortality and a steeper CFS-mortality gradient, the distributional imbalance becomes even more significant given the considerable impact of movements within frail strata (CFS 5 ↔ 7). The selected mortality thresholds of 2% and 5% are context-dependent and their clinical relevance vary related to baseline mortality, type of disease and intervention. The random sampling procedure is different from real-life study enrolment which may be susceptible to other objective and subjective factors. Some patients in the study cohorts might have been involved in actual clinical trials, although estimated to represent <5% of total, meaning that any investigated therapy would need to have an exceedingly high effect on mortality to sufficiently skew the outcome data which is considered implausible. The simulations quantify the extent to which frailty imbalance alone could theoretically generate apparent mortality differences based on the observed CFS-mortality relationship in the source cohort. The baseline data were used in the simulations without any additional modelling of assumed subsequent treatment effect(s). In the context of clinical trials, the results do not estimate treatment effect bias within the potential outcomes framework. The prevalence of CFS 8 was <1% and not explored further given that these patients are not expected to survive even minor illness and unlikely to be enrolled in clinical trials. The results should be viewed as hypothesis generating to stimulate debate and action on frailty as a design factor in clinical trials. Empirical analysis of published ICU trial data with available CFS distributions could quantify the actual prevalence of distributional imbalance in completed trials. The statistical codes are provided to facilitate such analyses.

## Conclusions

Baseline distributional imbalance in frailty assessed by the ordinal Clinical Frailty Scale may create clinically meaningful differences in mortality estimates. Studies reporting a balance between non-frail vs frail proportions while retaining ordinal distributional imbalance are still prone to residual confounding. While the findings in the study are hypothesis-generating, they suggest that intensive care trials should report complete CFS distributions, consider stratification by frailty in study designs, and adjust for ordinal CFS in analyses.

## Supplementary Information

Additional file 1.

## Data Availability

No datasets were generated or analysed during the current study.

## Abbreviations

CFS: Clinical frailty scale
SMD: Standardised mean difference
