# Supplementary material for: Simulation study of frailty as a baseline confounder: the need to improve reporting intensive care trials
Source: Crit Care. 2026 Jun 2;30:404. doi: 10.1186/s13054-026-06118-2 (PMC13445662; doi:10.1186/s13054-026-06118-2)
Supplement: Supplementary file 1 [file 13054_2026_6118_MOESM1_ESM.docx]

**Additional file 1**

**Simulation study of frailty as a baseline confounder: the need to improve reporting intensive care trials**

Christopher Dugan^1^, Suzanne Weightman^1^, Anders Aneman^1,2^

Table of Contents

[Calculations	2](#_Toc230091307)

[Table S1. Detailed threshold criteria	3](#_Toc230091308)

[Bidirectional CFS Threshold Calculation	6](#_Toc230091309)

[Description	6](#_Toc230091310)

[Requirements	6](#_Toc230091311)

[R Code	6](#_Toc230091312)

[Output Files	12](#_Toc230091313)

[Notes	12](#_Toc230091314)

[Paradoxical Scenario Simulation	13](#_Toc230091315)

[Description	13](#_Toc230091316)

[Requirements	13](#_Toc230091317)

[Input Requirements	13](#_Toc230091318)

[R Code	13](#_Toc230091319)

[Output Files	17](#_Toc230091320)

[Notes	17](#_Toc230091321)

[Simulation using permuted block randomisation	17](#_Toc230091322)

[Requirements	17](#_Toc230091323)

[Input Data Requirements	17](#_Toc230091324)

[R Code	18](#_Toc230091325)

[Output Files	26](#_Toc230091326)

[STROBE checklist	26](#_Toc230091327)

## Calculations

(1) The standardised mean difference (SMD) in binary CFS distributions in ‘trials’ of different sizes was calculated as:

$$Standardised Mean Difference (SMD)= \frac{(pcontrol-pintervention)}{\sqrt{ppooled \times(1-ppooled)}}$$

where p_control_ is the proportion frail patients in the control arm, p_intervention_ is the proportion frail patients in the intervention arm and p_pooled_ is the pooled proportion frail patients among the n number of patients calculated as:

$$ppooled= \frac{(ncontrol \times pcontrol +nintervention \times pintervention)}{(ncontrol +nintervention)}$$

(2) The mean distribution of CFS in each of the two ‘trial’ arms was calculated as:

$$mean CFS= \frac{\sum\left( CFSi \times ni \right)}{N}$$

where CFS_i_ and n_i_ represent the counts in individual strata CFS 1-8 among a total sample size of N randomly selected patients. The absolute mean CFS difference was calculated as:

$$\left| mean CFS difference \right|= meanCFScontrol -meanCFSintervention$$

(3) The population attributable fraction (PAF) was calculated using the equation:

$$Population Attributable Fraction= \frac{p \times(RR-1)}{1+p \times(RR-1)}$$

where p is the prevalence of frailty (CFS ≥5) and RR is the relative risk of in-hospital death (non-frail vs frail). Death attributable to frailty was then calculated as the observed mortality times the population attributable fraction.

Table S1. Detailed threshold criteria

The table below shows the detailed threshold criteria for clinical frailty scale (CFS) imbalance to generate differences in observed in-hospital mortality of 1%, 2% ,3% and 5% in the different diagnostic groups.

| GROUP | Mortality difference | CFS shift | % shift | Mean CFS change |
| --- | --- | --- | --- | --- |
| Critically ill | 1% | 1-4 ↔ 5 | 22.2 | 0.43 |
| Critically ill | 1% | 1-4 ↔ 6 | 9.65 | 0.28 |
| Critically ill | 1% | 1-4 ↔ 7 | 4.69 | 0.19 |
| Critically ill | 1% | 5 ↔ 6 | 17.0 | 0.17 |
| Critically ill | 1% | 5 ↔ 7 | 5.95 | 0.12 |
| Critically ill | 1% | 6 ↔7 | 9.14 | 0.09 |
| Critically ill | 2% | 1-4 ↔ 5 | 44.5 | 0.87 |
| Critically ill | 2% | 1-4 ↔ 6 | 19.4 | 0.57 |
| Critically ill | 2% | 1-4 ↔ 7 | 9.39 | 0.37 |
| Critically ill | 2% | 5 ↔ 6 | 34.1 | 0.34 |
| Critically ill | 2% | 5 ↔ 7 | 11.9 | 0.24 |
| Critically ill | 2% | 6 ↔ 7 | 18.3 | 0.18 |
| Critically ill | 3% | 1-4 ↔ 5 | 66.7 | 1.30 |
| Critically ill | 3% | 1-4 ↔ 6 | 28.9 | 0.85 |
| Critically ill | 3% | 1-4 ↔ 7 | 14.1 | 0.56 |
| Critically ill | 3% | 5 ↔ 6 | 51.1 | 0.51 |
| Critically ill | 3% | 5 ↔ 7 | 17.9 | 0.36 |
| Critically ill | 3% | 6 ↔ 7 | 27.4 | 0.27 |
| Critically ill | 5% | 1-4 ↔ 5 | 65.3 | 2.17 |
| Critically ill | 5% | 1-4 ↔ 6 | 48.2 | 1.42 |
| Critically ill | 5% | 1-4 ↔ 7 | 23.5 | 0.93 |
| Critically ill | 5% | 5 ↔ 6 | 85.2 | 0.85 |
| Critically ill | 5% | 5 ↔ 7 | 29.6 | 0.59 |
| Critically ill | 5% | 6 ↔ 7 | 45.7 | 0.46 |
| Sepsis | 1% | 1-4 ↔ 5 | 100 | 1.77 |
| Sepsis | 1% | 1-4 ↔ 6 | 9.32 | 0.26 |
| Sepsis | 1% | 1-4 ↔ 7 | 5.78 | 0.22 |
| Sepsis | 1% | 5 ↔ 6 | 10.3 | 0.10 |
| Sepsis | 1% | 5 ↔ 7 | 6.13 | 0.12 |
| Sepsis | 1% | 6 ↔ 7 | 15.2 | 0.15 |
| Sepsis | 2% | 1-4 ↔ 5 | 34.3 | 1.55 |
| Sepsis | 2% | 1-4 ↔ 6 | 18.6 | 0.52 |
| Sepsis | 2% | 1-4 ↔ 7 | 11.6 | 0.44 |
| Sepsis | 2% | 5 ↔ 6 | 20.6 | 0.21 |
| Sepsis | 2% | 5 ↔ 7 | 12.3 | 0.25 |
| Sepsis | 2% | 6 ↔ 7 | 30.4 | 0.30 |
| Sepsis | 3% | 1-4 ↔ 5 | 300 | 5.32 |
| Sepsis | 3% | 1-4 ↔ 6 | 28.0 | 0.77 |
| Sepsis | 3% | 1-4 ↔ 7 | 17.3 | 0.65 |
| Sepsis | 3% | 5 ↔ 6 | 30.8 | 0.31 |
| Sepsis | 3% | 5 ↔ 7 | 18.4 | 0.37 |
| Sepsis | 3% | 6 ↔ 7 | 45.7 | 0.46 |
| Sepsis | 5% | 1-4 ↔ 5 | 64.9 | 2.87 |
| Sepsis | 5% | 1-4 ↔ 6 | 46.6 | 1.29 |
| Sepsis | 5% | 1-4 ↔ 7 | 28.9 | 1.09 |
| Sepsis | 5% | 5 ↔ 6 | 51.4 | 0.51 |
| Sepsis | 5% | 5 ↔ 7 | 30.7 | 0.61 |
| Sepsis | 5% | 6 ↔ 7 | 76.1 | 0.76 |
| Acute respiratory failure | 1% | 1-4 ↔ 5 | 17.0 | 0.33 |
| Acute respiratory failure | 1% | 1-4 ↔ 6 | 9.03 | 0.26 |
| Acute respiratory failure | 1% | 1-4 ↔ 7 | 4.00 | 0.16 |
| Acute respiratory failure | 1% | 5 ↔ 6 | 19.2 | 0.19 |
| Acute respiratory failure | 1% | 5 ↔ 7 | 5.23 | 0.10 |
| Acute respiratory failure | 1% | 6 ↔ 7 | 7.19 | 0.07 |
| Acute respiratory failure | 2% | 1-4 ↔ 5 | 34.0 | 0.66 |
| Acute respiratory failure | 2% | 1-4 ↔ 6 | 18.1 | 0.53 |
| Acute respiratory failure | 2% | 1-4 ↔ 7 | 8.00 | 0.31 |
| Acute respiratory failure | 2% | 5 ↔ 6 | 38.5 | 0.38 |
| Acute respiratory failure | 2% | 5 ↔ 7 | 10.5 | 0.21 |
| Acute respiratory failure | 2% | 6 ↔ 7 | 14.4 | 0.14 |
| Acute respiratory failure | 3% | 1-4 ↔ 5 | 51.0 | 0.99 |
| Acute respiratory failure | 3% | 1-4 ↔ 6 | 27.1 | 0.79 |
| Acute respiratory failure | 3% | 1-4 ↔ 7 | 12.0 | 0.47 |
| Acute respiratory failure | 3% | 5 ↔ 6 | 57.7 | 0.58 |
| Acute respiratory failure | 3% | 5 ↔ 7 | 15.7 | 0.31 |
| Acute respiratory failure | 3% | 6 ↔ 7 | 21.6 | 0.22 |
| Acute respiratory failure | 5% | 1-4 ↔ 5 | 85.0 | 1.64 |
| Acute respiratory failure | 5% | 1-4 ↔ 6 | 45.1 | 1.32 |
| Acute respiratory failure | 5% | 1-4 ↔ 7 | 20.0 | 0.79 |
| Acute respiratory failure | 5% | 5 ↔ 6 | 96.2 | 0.96 |
| Acute respiratory failure | 5% | 5 ↔ 7 | 26.2 | 0.52 |
| Acute respiratory failure | 5% | 6 ↔ 7 | 35.9 | 0.36 |
| Shock | 1% | 1-4 ↔ 5 | 61.6 | 1.27 |
| Shock | 1% | 1-4 ↔ 6 | 13.2 | 0.41 |
| Shock | 1% | 1-4 ↔ 7 | 7.41 | 0.30 |
| Shock | 1% | 5 ↔ 6 | 10.9 | 0.11 |
| Shock | 1% | 5 ↔ 7 | 6.62 | 0.13 |
| Shock | 1% | 6 ↔ 7 | 16.9 | 0.17 |
| Shock | 2% | 1-4 ↔ 5 | 52.3 | 1.54 |
| Shock | 2% | 1-4 ↔ 6 | 26.5 | 0.81 |
| Shock | 2% | 1-4 ↔ 7 | 14.8 | 0.60 |
| Shock | 2% | 5 ↔ 6 | 21.8 | 0.22 |
| Shock | 2% | 5 ↔ 7 | 13.2 | 0.26 |
| Shock | 2% | 6 ↔ 7 | 33.7 | 0.34 |
| Shock | 3% | 1-4 ↔ 5 | 78.5 | 3.81 |
| Shock | 3% | 1-4 ↔ 6 | 39.7 | 1.22 |
| Shock | 3% | 1-4 ↔ 7 | 22.2 | 0.90 |
| Shock | 3% | 5 ↔ 6 | 32.7 | 0.33 |
| Shock | 3% | 5 ↔ 7 | 19.6 | 0.40 |
| Shock | 3% | 6 ↔ 7 | 50.6 | 0.51 |
| Shock | 5% | 1-4 ↔ 5 | 76.8 | 2.35 |
| Shock | 5% | 1-4 ↔ 6 | 66.1 | 2.03 |
| Shock | 5% | 1-4 ↔ 7 | 37.1 | 1.51 |
| Shock | 5% | 5 ↔ 6 | 54.5 | 0.54 |
| Shock | 5% | 5 ↔ 7 | 33.1 | 0.66 |
| Shock | 5% | 6 ↔ 7 | 84.3 | 0.84 |

## Bidirectional CFS Threshold Calculation

### Description

This R script calculates data-driven thresholds for Clinical Frailty Scale (CFS) distributional imbalance that correspond to specific mortality differences. The analysis is bidirectional, accounting for shifts in both directions (less frail → more frail AND more frail → less frail).

### Requirements

**Required R packages:** tidyverse, readxl

Input Data Requirements

Your dataset (as an MS Excel file) must contain:

• CFS: Clinical Frailty Scale score (numeric 1-8)

• Hospital mortality indicator (0 = survived, 1 = died)

• Diagnostic category indicators (if analysing by subgroup)

**Note:** Modify variable names in lines 36-42 to match your dataset.

### R Code

**Instructions:** (1) Update the file path on line 32 to your data file, (2) Modify variable names on lines 36-42 to match your dataset, (3) Define cohorts on lines 45-51 as needed for your analysis.

```
#=======================================================================
# OBJECTIVE THRESHOLDS BY DIAGNOSTIC CATEGORY (BIDIRECTIONAL)
#=======================================================================

library(tidyverse)
library(readxl)
set.seed(123)

#======================================================================
# LOAD YOUR DATA
#=======================================================================

# UPDATE THIS PATH to your data file
file_path <- "path/to/your/data.xlsx"
df_raw <- read_excel(file_path)

# MODIFY THESE variable names to match your dataset
df <- df_raw %>%
  mutate(
    cfs_score = as.numeric(YOUR_CFS_VARIABLE),
    hospital_mortality = YOUR_MORTALITY_VARIABLE,  # 1 = died, 0 = survived
    frailty_binary = if_else(cfs_score >= 5, 1, 0)
  ) %>%
  filter(!is.na(cfs_score) & !is.na(hospital_mortality))

# DEFINE YOUR COHORTS (modify as needed)
# Example: analyzing entire cohort
cohorts <- list(
  Combined = df  # Use your entire cohort
  # Add diagnostic subgroups if needed:
  # Subgroup1 = df %>% filter(YOUR_CONDITION),
  # Subgroup2 = df %>% filter(YOUR_CONDITION)
)

#======================================================================
# CALCULATE CFS-MORTALITY RELATIONSHIP
#======================================================================

cohort_mortality_data <- list()

for(cohort_name in names(cohorts)) {

  cohort_df <- cohorts[[cohort_name]]

  # Calculate mortality by CFS (excluding CFS 8 - typically too rare)
  cfs_mortality <- cohort_df %>%
    filter(cfs_score <= 7) %>%
    group_by(cfs_score) %>%
    summarise(
      n = n(),
      deaths = sum(hospital_mortality),
      mortality_rate = mean(hospital_mortality),
      .groups = "drop"
    )

  # Calculate average mortality for CFS 1-4
  mort_1_4 <- cfs_mortality %>%
    filter(cfs_score <= 4) %>%
    summarise(avg_mort = weighted.mean(mortality_rate, n)) %>%
    pull(avg_mort)

  # Individual CFS 5-7 mortality rates
  mort_5 <- cfs_mortality %>% filter(cfs_score == 5) %>% pull(mortality_rate)
  mort_6 <- cfs_mortality %>% filter(cfs_score == 6) %>% pull(mortality_rate)
  mort_7 <- cfs_mortality %>% filter(cfs_score == 7) %>% pull(mortality_rate)

  # Handle missing strata
  if(length(mort_5) == 0) mort_5 <- NA
  if(length(mort_6) == 0) mort_6 <- NA
  if(length(mort_7) == 0) mort_7 <- NA

  # Store data
  cohort_mortality_data[[cohort_name]] <- list(
    data = cfs_mortality,
    mort_1_4 = mort_1_4,
    mort_5 = mort_5,
    mort_6 = mort_6,
    mort_7 = mort_7
  )
}

#======================================================================
# CALCULATE BIDIRECTIONAL THRESHOLDS
#======================================================================

# Function: Calculate shift needed (BIDIRECTIONAL)
calculate_shift_for_target <- function(target_mort_diff, mort_from, mort_to) {
  if(is.na(mort_to) | is.na(mort_from)) return(NA)

  mort_impact_per_pct <- (mort_to - mort_from) / 100
  if(mort_impact_per_pct == 0) return(NA)

  pct_shift <- target_mort_diff / mort_impact_per_pct
  return(abs(pct_shift))  # Return absolute value
}

# Function: Calculate mean CFS change (BIDIRECTIONAL)
calculate_mean_cfs_change <- function(pct_shift, cfs_from_avg, cfs_to) {
  if(is.na(pct_shift)) return(NA)
  delta <- (pct_shift / 100) * (cfs_to - cfs_from_avg)
  return(abs(delta))  # Return absolute value
}

# Target mortality differences to analyze
targets <- c(0.01, 0.02, 0.03, 0.05)  # 1%, 2%, 3%, 5%
target_labels <- c("1%", "2%", "3%", "5%")

threshold_summary <- tibble()
detailed_shifts <- tibble()

for(cohort_name in names(cohort_mortality_data)) {

  cdata <- cohort_mortality_data[[cohort_name]]

  # Average CFS in 1-4 (weighted)
  cfs_1_4_data <- cdata$data %>% filter(cfs_score <= 4)
  avg_cfs_1_4 <- weighted.mean(cfs_1_4_data$cfs_score, cfs_1_4_data$n)

  for(i in seq_along(targets)) {
    target <- targets[i]
    target_label <- target_labels[i]

    #===================================================================
    # UPWARD SHIFTS (less frail → more frail)
    #===================================================================

    shift_1_4_to_5 <- calculate_shift_for_target(target, cdata$mort_1_4, cdata$mort_5)
    shift_1_4_to_6 <- calculate_shift_for_target(target, cdata$mort_1_4, cdata$mort_6)
    shift_1_4_to_7 <- calculate_shift_for_target(target, cdata$mort_1_4, cdata$mort_7)
    shift_5_to_6 <- calculate_shift_for_target(target, cdata$mort_5, cdata$mort_6)
    shift_5_to_7 <- calculate_shift_for_target(target, cdata$mort_5, cdata$mort_7)
    shift_6_to_7 <- calculate_shift_for_target(target, cdata$mort_6, cdata$mort_7)

    #===================================================================
    # DOWNWARD SHIFTS (more frail → less frail)
    #===================================================================

    shift_5_to_1_4 <- calculate_shift_for_target(target, cdata$mort_5, cdata$mort_1_4)
    shift_6_to_1_4 <- calculate_shift_for_target(target, cdata$mort_6, cdata$mort_1_4)
    shift_7_to_1_4 <- calculate_shift_for_target(target, cdata$mort_7, cdata$mort_1_4)
    shift_6_to_5 <- calculate_shift_for_target(target, cdata$mort_6, cdata$mort_5)
    shift_7_to_5 <- calculate_shift_for_target(target, cdata$mort_7, cdata$mort_5)
    shift_7_to_6 <- calculate_shift_for_target(target, cdata$mort_7, cdata$mort_6)

    # Calculate mean CFS changes
    mean_cfs_1_4_to_5 <- calculate_mean_cfs_change(shift_1_4_to_5, avg_cfs_1_4, 5)
    mean_cfs_1_4_to_6 <- calculate_mean_cfs_change(shift_1_4_to_6, avg_cfs_1_4, 6)
    mean_cfs_1_4_to_7 <- calculate_mean_cfs_change(shift_1_4_to_7, avg_cfs_1_4, 7)
    mean_cfs_5_to_6 <- calculate_mean_cfs_change(shift_5_to_6, 5, 6)
    mean_cfs_5_to_7 <- calculate_mean_cfs_change(shift_5_to_7, 5, 7)
    mean_cfs_6_to_7 <- calculate_mean_cfs_change(shift_6_to_7, 6, 7)
    mean_cfs_5_to_1_4 <- calculate_mean_cfs_change(shift_5_to_1_4, 5, avg_cfs_1_4)
    mean_cfs_6_to_1_4 <- calculate_mean_cfs_change(shift_6_to_1_4, 6, avg_cfs_1_4)
    mean_cfs_7_to_1_4 <- calculate_mean_cfs_change(shift_7_to_1_4, 7, avg_cfs_1_4)
    mean_cfs_6_to_5 <- calculate_mean_cfs_change(shift_6_to_5, 6, 5)
    mean_cfs_7_to_5 <- calculate_mean_cfs_change(shift_7_to_5, 7, 5)
    mean_cfs_7_to_6 <- calculate_mean_cfs_change(shift_7_to_6, 7, 6)

    # Compile all shifts (BIDIRECTIONAL)
    all_shifts <- tibble(
      shift_type = c(
        "1-4 → 5", "1-4 → 6", "1-4 → 7",
        "5 → 6", "5 → 7", "6 → 7",
        "5 → 1-4", "6 → 1-4", "7 → 1-4",
        "6 → 5", "7 → 5", "7 → 6"
      ),
      pct_shift = c(
        shift_1_4_to_5, shift_1_4_to_6, shift_1_4_to_7,
        shift_5_to_6, shift_5_to_7, shift_6_to_7,
        shift_5_to_1_4, shift_6_to_1_4, shift_7_to_1_4,
        shift_6_to_5, shift_7_to_5, shift_7_to_6
      ),
      mean_cfs_change = c(
        mean_cfs_1_4_to_5, mean_cfs_1_4_to_6, mean_cfs_1_4_to_7,
        mean_cfs_5_to_6, mean_cfs_5_to_7, mean_cfs_6_to_7,
        mean_cfs_5_to_1_4, mean_cfs_6_to_1_4, mean_cfs_7_to_1_4,
        mean_cfs_6_to_5, mean_cfs_7_to_5, mean_cfs_7_to_6
      ),
      direction = c(
        rep("Upward", 6),
        rep("Downward", 6)
      )
    ) %>%
      filter(!is.na(pct_shift))

    if(nrow(all_shifts) > 0) {

      # Find minimum threshold
      min_shift_row <- all_shifts %>% slice_min(pct_shift, n = 1) %>% slice(1)

      # Store summary
      threshold_summary <- bind_rows(threshold_summary, tibble(
        cohort = cohort_name,
        target_mort_diff = target_label,
        min_pct_shift = min_shift_row$pct_shift[1],
        min_mean_cfs_change = min_shift_row$mean_cfs_change[1],
        shift_type = min_shift_row$shift_type[1]
      ))

      # Store all detailed shifts
      for(k in 1:nrow(all_shifts)) {
        detailed_shifts <- bind_rows(detailed_shifts, tibble(
          cohort = cohort_name,
          target_mort_diff = target_label,
          shift_type = all_shifts$shift_type[k],
          direction = all_shifts$direction[k],
          pct_shift = all_shifts$pct_shift[k],
          mean_cfs_change = all_shifts$mean_cfs_change[k]
        ))
      }
    }
  }
}

#=======================================================================
# SAVE RESULTS
#=======================================================================

write.csv(threshold_summary, "thresholds_summary_BIDIRECTIONAL.csv",
          row.names = FALSE)
write.csv(detailed_shifts, "detailed_shifts_BIDIRECTIONAL.csv",
          row.names = FALSE)
```

### Output Files

**1. thresholds_summary_BIDIRECTIONAL.csv:** Summary of minimum mean CFS change thresholds for each mortality target

**2. detailed_shifts_BIDIRECTIONAL.csv:** Complete listing of all calculated shifts (upward and downward) with percentage shifts and mean CFS changes

### Notes

The *min_mean_cfs_change* column in the thresholds_summary file represents the minimum absolute difference in mean CFS between two groups that would create the specified mortality difference.

**Bidirectionality:** The analysis detects imbalance regardless of which trial arm has higher mean CFS, to comprehensively identify distributional confounding.

## Paradoxical Scenario Simulation

### Description

This R script simulates trials to identify paradoxical scenarios defined as trials that achieve binary balance (SMD < 0.1 for frailty [CFS1-4] prevalence) but exhibit distributional imbalance in the Clinical Frailty Scale. The script uses the data-driven bidirectional thresholds from Supplementary Code 1 that must be run first.

### Requirements

**Required R packages:** tidyverse, readxl

### Input Requirements

**1. Dataset:** Same requirements as Supplementary Code 1

**2. Threshold file:** detailed_shifts_BIDIRECTIONAL.csv (output from Supplementary Code 1)

### R Code

**Instructions:** (1) Run Supplementary Code 1 first to generate thresholds, (2) Update file paths on lines 14 and 33, (3) Modify variable names on lines 18-22 to match your dataset.

```
#=======================================================================
# PARADOXICAL SCENARIO SIMULATION
#=======================================================================

library(tidyverse)
library(readxl)
set.seed(123)

#=======================================================================
# LOAD YOUR DATA
#=======================================================================

# UPDATE THIS PATH to your data file
file_path <- "path/to/your/data.xlsx"
df_raw <- read_excel(file_path)

# MODIFY THESE variable names to match your dataset
df <- df_raw %>%
  mutate(
    cfs_score = as.numeric(YOUR_CFS_VARIABLE),
    hospital_mortality = YOUR_MORTALITY_VARIABLE,
    frailty_binary = if_else(cfs_score >= 5, 1, 0)
  ) %>%
  filter(!is.na(cfs_score) & !is.na(hospital_mortality), cfs_score <= 7)

# DEFINE YOUR COHORT (modify as needed)
cohorts <- list(
  Combined = df
)

#=======================================================================
# LOAD THRESHOLDS
#=======================================================================

# UPDATE THIS PATH to your threshold file
threshold_file <- "path/to/detailed_shifts_BIDIRECTIONAL.csv"
thresholds_raw <- read.csv(threshold_file)

# Extract 2% mortality thresholds
cohort_thresholds <- thresholds_raw %>%
  filter(target_mort_diff == "2%") %>%
  group_by(cohort) %>%
  summarise(
    threshold_mean_cfs = min(mean_cfs_change, na.rm = TRUE),
    .groups = "drop"
  )

#=======================================================================
# SIMULATION PARAMETERS
#=======================================================================

N_TRIALS <- 100
SAMPLE_SIZES <- c(100, 150, 200, 250, 300, 400, 500, 750, 1000)

#=======================================================================
# SIMULATION FUNCTION
#=======================================================================

simulate_trial <- function(cohort_data, cohort_name, trial_id,
                           n_per_arm, threshold_mean_cfs) {

  trial_sample <- cohort_data %>%
    slice_sample(n = n_per_arm * 2, replace = TRUE) %>%
    mutate(arm = sample(rep(c("Control", "Intervention"), each = n_per_arm)))

  control <- trial_sample %>% filter(arm == "Control")
  intervention <- trial_sample %>% filter(arm == "Intervention")

  mean_cfs_control <- mean(control$cfs_score)
  mean_cfs_intervention <- mean(intervention$cfs_score)
  mean_cfs_diff <- abs(mean_cfs_intervention - mean_cfs_control)

  p_control_frail <- mean(control$frailty_binary)
  p_intervention_frail <- mean(intervention$frailty_binary)
  pooled_p <- mean(trial_sample$frailty_binary)

  if(pooled_p > 0 & pooled_p < 1) {
    binary_smd <- abs(p_intervention_frail - p_control_frail) /
      sqrt(pooled_p * (1 - pooled_p))
  } else {
    binary_smd <- 0
  }

  binary_balanced <- binary_smd < 0.1
  distributionally_imbalanced <- mean_cfs_diff >= threshold_mean_cfs
  paradoxical <- distributionally_imbalanced & binary_balanced

  tibble(
    trial_id = trial_id,
    cohort = cohort_name,
    n_per_arm = n_per_arm,
    mean_cfs_diff = mean_cfs_diff,
    binary_smd = binary_smd,
    binary_balanced = binary_balanced,
    distributionally_imbalanced = distributionally_imbalanced,
    paradoxical = paradoxical,
    threshold_used = threshold_mean_cfs
  )
}

#=======================================================================
# RUN SIMULATIONS
#=======================================================================

all_simulated_trials <- tibble()

for(cohort_name in names(cohorts)) {
  cohort_data <- cohorts[[cohort_name]]
  threshold <- cohort_thresholds %>%
    filter(cohort == cohort_name) %>%
    pull(threshold_mean_cfs)

  if(length(threshold) == 0) next

  for(n_per_arm in SAMPLE_SIZES) {
    for(trial_id in 1:N_TRIALS) {
      trial_result <- simulate_trial(
        cohort_data, cohort_name, trial_id, n_per_arm, threshold
      )
      all_simulated_trials <- bind_rows(all_simulated_trials, trial_result)
    }
  }
}

#=======================================================================
# SAVE RESULTS
#=======================================================================

paradox_summary <- all_simulated_trials %>%
  group_by(cohort, n_per_arm) %>%
  summarise(
    n_trials = n(),
    n_paradoxical = sum(paradoxical),
    pct_paradoxical = (n_paradoxical / n_trials) * 100,
    .groups = "drop"
  )

write.csv(all_simulated_trials, "simulated_trials_BIDIRECTIONAL.csv", row.names = FALSE)
write.csv(paradox_summary, "paradox_summary_BIDIRECTIONAL.csv", row.names = FALSE)
```

### Output Files

**1. simulated_trials_BIDIRECTIONAL.csv:** Complete trial-level data for all simulations

**2. paradox_summary_BIDIRECTIONAL.csv:** Summary statistics by cohort and sample size

### Notes

**Paradoxical scenario prevalence (pct_paradoxical):** Percentage of trials that passed binary balance (SMD < 0.1) but exhibited distributional imbalance exceeding the cohort-specific threshold.

**Bidirectionality:** Analysis detects imbalance regardless of which trial arm has higher mean CFS, to comprehensively identify distributional confounding.

## Simulation using permuted block randomisation

This R script simulates randomized controlled trials using permuted block randomisation to compare with simple 1:1 randomization. It evaluates whether block randomization reduces the prevalence of paradoxical trials (trials achieving binary frailty balance but retaining distributional imbalance sufficient to create ≥2% mortality difference).

The script uses objective thresholds calculated from observed CFS-mortality relationships to classify trials as distributionally imbalanced or paradoxical.

### Requirements

**Required R packages:** tidyverse, readxl

### Input Data Requirements

Patient-level dataset must contain:

• CFS: Clinical Frailty Scale score (numeric 1-8) • Hospital mortality indicator (0 = survived, 1 = died) • Diagnostic category indicators (shock, sepsis, ARDS)

Threshold file required:

• CSV file containing pre-calculated thresholds from bidirectional threshold analysis • Must contain columns: cohort, target_mort_diff, mean_cfs_change, pct_shift, shift_type • Generated by Supplementary Code 1 (Threshold Calculation script)

**Note:** Modify file paths on lines 74 and 99 to match your data locations. Modify variable names on lines 78-86 to match your dataset.

Key Features

1. **Permuted block randomization function** - Generates randomization sequences with block sizes 4, 6, or 8
2. **Multiple block sizes tested** - Compares effectiveness across different block configurations
3. **Paradoxical trial detection** - Identifies trials with binary balance but distributional imbalance
4. **Comparison with simple randomization** - Assesses whether blocking provides protection

### R Code

Instructions:

(1) Update file path on line 74 to your patient data file
(2) Update file path on line 99 to your threshold file (from Supplementary Code 1)
(3) Modify variable names on lines 78-86 to match your dataset
(4) Define diagnostic cohorts on lines 89-94 as needed
(5) Choose block sizes to test on line 108 (default: 4, 6, 8)

\# ============================================================================
\# PARADOXICAL TRIAL SIMULATION - PERMUTED BLOCK RANDOMIZATION VERSION
\# ============================================================================
\#
\# Compares simple 1:1 randomization with permuted block randomization
\# to assess whether blocking reduces paradoxical trial prevalence
\#
\# ============================================================================

library(tidyverse)
library(readxl)

\# ============================================================================
\# PERMUTED BLOCK RANDOMIZATION FUNCTION
\# ============================================================================

generate_permuted_blocks <- function(n_per_arm, block_size = 4) {
 #' Generate permuted block randomization sequence
 #' @param n_per_arm Number per arm (total n = 2 × n_per_arm)
 #' @param block_size Block size (must be even, typically 4, 6, or 8)
 #' @return Vector of "Control" and "Intervention" allocations

 total_n <- n_per_arm * 2

 \# Validate
 if(block_size %% 2 != 0) stop("block_size must be even")

 \# Calculate number of complete blocks
 n_complete_blocks <- total_n %/% block_size
 remainder <- total_n %% block_size

 \# Build sequence
 allocation_sequence <- c()

 \# Add complete blocks
 for(i in 1:n_complete_blocks) {
 \# Create block: half Control, half Intervention
 block <- c(rep("Control", block_size/2),
 rep("Intervention", block_size/2))
 \# Randomly shuffle within block
 permuted_block <- sample(block)
 \# Add to sequence
 allocation_sequence <- c(allocation_sequence, permuted_block)
 }

 \# Handle remainder (if any)
 if(remainder > 0 && remainder %% 2 == 0) {
 block <- c(rep("Control", remainder/2),
 rep("Intervention", remainder/2))
 permuted_block <- sample(block)
 allocation_sequence <- c(allocation_sequence, permuted_block)
 }

 return(allocation_sequence)
}

set.seed(123)

cat("\n══════════════════════════════════════════════════════════════════\n")
cat("BLOCK RANDOMIZATION SIMULATION\n")
cat("══════════════════════════════════════════════════════════════════\n\n")

\# ============================================================================
\# LOAD DATA
\# ============================================================================

cat("Loading patient data...\n")

\# MODIFY THIS PATH to your data file
file_path <- "path/to/your/data.xlsx"
df_raw <- read_excel(file_path)

\# MODIFY variable names to match your dataset
df <- df_raw %>%
 mutate(
 shock = as.numeric(SHOCK),
 sepsis = as.numeric(SEPSIS3),
 ards = as.numeric(ARDS),
 cfs_score = as.numeric(CFS),
 frailty_binary = if_else(cfs_score >= 5, 1, 0),
 hospital_mortality = if_else(as.numeric(SURVHOSP) == 0, 1, 0)
 ) %>%
 filter(!is.na(cfs_score) & !is.na(hospital_mortality), cfs_score <= 7)

\# MODIFY cohort definitions as needed for your analysis
cohorts <- list(
 Combined = df %>% filter(shock == 1 | sepsis == 1 | ards == 1),
 Sepsis = df %>% filter(sepsis == 1),
 ARDS = df %>% filter(ards == 1),
 Shock = df %>% filter(shock == 1)
)

\# MODIFY THIS PATH to your threshold file
threshold_file <- "path/to/your/detailed_shifts_by_cohort.csv"
thresholds_raw <- read.csv(threshold_file)

cat(" ✓ Data loaded successfully\n\n")

\# ============================================================================
\# SIMULATION PARAMETERS
\# ============================================================================

\# MODIFY these parameters as needed
N_TRIALS <- 100	# Number of trials to simulate per scenario
SAMPLE_SIZES <- c(100, 200, 250, 300, 400, 500, 750, 1000) # Per arm
BLOCK_SIZES <- c(4, 6, 8)	# Block sizes to test

cat("Simulation parameters:\n")
cat(" Trials per scenario: ", N_TRIALS, "\n")
cat(" Sample sizes (per arm): ", paste(SAMPLE_SIZES, collapse = ", "), "\n")
cat(" Block sizes tested: ", paste(BLOCK_SIZES, collapse = ", "), "\n\n")

\# ============================================================================
\# EXTRACT THRESHOLDS
\# ============================================================================

\# Get threshold for 2% mortality difference (minimum across shift types)
cohort_thresholds <- thresholds_raw %>%
 filter(target_mort_diff == "2%") %>%
 group_by(cohort) %>%
 summarise(
 threshold_mean_cfs = min(abs(mean_cfs_change), na.rm = TRUE),
 .groups = "drop"
 )

cat("Thresholds for 2% mortality difference:\n")
print(cohort_thresholds)
cat("\n")

\# ============================================================================
\# WILSON SCORE CONFIDENCE INTERVAL FUNCTION
\# ============================================================================

calc_proportion_ci <- function(x, n) {
 #' Calculate Wilson score confidence interval for proportion
 #' @param x Number of successes
 #' @param n Total number of trials
 #' @return List with lower and upper bounds (as percentages)

 p <- x / n
 z <- 1.96 # 95% CI

 denominator <- 1 + z^2 / n
 centre <- (p + z^2 / (2 * n)) / denominator
 spread <- z * sqrt(p * (1 - p) / n + z^2 / (4 * n^2)) / denominator

 lower <- centre - spread
 upper <- centre + spread

 return(list(lower = max(0, lower) * 100, upper = min(1, upper) * 100))
}

\# ============================================================================
\# TRIAL SIMULATION FUNCTION
\# ============================================================================

simulate_blocked_trial <- function(cohort_data, cohort_name, trial_id,
 n_per_arm, threshold_mean_cfs,
 block_size = NULL) {
 #' Simulate one trial with specified randomization method
 #' @param cohort_data Patient data for this cohort
 #' @param cohort_name Name of diagnostic cohort
 #' @param trial_id Unique trial identifier
 #' @param n_per_arm Sample size per arm
 #' @param threshold_mean_cfs Threshold for distributional imbalance
 #' @param block_size If NULL, use simple 1:1; otherwise use blocked

 \# Sample patients with replacement
 trial_sample <- cohort_data %>%
 slice_sample(n = n_per_arm * 2, replace = TRUE)

 \# Assign treatment allocation
 if(is.null(block_size)) {
 \# Simple 1:1 randomization
 trial_sample <- trial_sample %>%
 mutate(arm = sample(rep(c("Control", "Intervention"), each = n_per_arm)))
 randomization_method <- "Simple 1:1"
 } else {
 \# Permuted block randomization
 allocation <- generate_permuted_blocks(n_per_arm, block_size)
 trial_sample <- trial_sample %>%
 mutate(arm = allocation)
 randomization_method <- paste0("Block size ", block_size)
 }

 \# Split into arms
 control <- trial_sample %>% filter(arm == "Control")
 intervention <- trial_sample %>% filter(arm == "Intervention")

 \# Calculate distributional metrics
 mean_cfs_control <- mean(control$cfs_score)
 mean_cfs_intervention <- mean(intervention$cfs_score)
 mean_cfs_diff <- abs(mean_cfs_intervention - mean_cfs_control)

 \# Binary balance check
 p_control_frail <- mean(control$frailty_binary)
 p_intervention_frail <- mean(intervention$frailty_binary)
 pooled_p <- mean(trial_sample$frailty_binary)

 if(pooled_p > 0 & pooled_p < 1) {
 binary_smd <- abs(p_intervention_frail - p_control_frail) /
 sqrt(pooled_p * (1 - pooled_p))
 } else {
 binary_smd <- 0
 }

 binary_balanced <- binary_smd < 0.1

 \# Distributional imbalance using objective threshold
 distributionally_imbalanced <- mean_cfs_diff >= threshold_mean_cfs

 \# Paradoxical trial
 paradoxical <- distributionally_imbalanced & binary_balanced

 tibble(
 trial_id = trial_id,
 cohort = cohort_name,
 n_per_arm = n_per_arm,
 randomization_method = randomization_method,
 block_size = ifelse(is.null(block_size), NA, block_size),
 mean_cfs_diff = mean_cfs_diff,
 binary_smd = binary_smd,
 binary_balanced = binary_balanced,
 distributionally_imbalanced = distributionally_imbalanced,
 paradoxical = paradoxical,
 threshold_used = threshold_mean_cfs
 )
}

\# ============================================================================
\# RUN SIMULATIONS
\# ============================================================================

cat("Running simulations...\n\n")

all_results <- tibble()

for(cohort_name in names(cohorts)) {

 cat("Processing cohort: ", cohort_name, "\n")

 cohort_df <- cohorts[[cohort_name]]
 threshold <- cohort_thresholds %>%
 filter(cohort == cohort_name) %>%
 pull(threshold_mean_cfs)

 for(n in SAMPLE_SIZES) {

 cat(" Sample size n=", n, " per arm\n", sep = "")

 \# Simple 1:1 randomization
 cat("	- Simple 1:1 randomization... ")
 simple_results <- map_dfr(1:N_TRIALS, ~simulate_blocked_trial(
 cohort_data = cohort_df,
 cohort_name = cohort_name,
 trial_id = paste0(cohort_name, "_n", n, "_simple_", .x),
 n_per_arm = n,
 threshold_mean_cfs = threshold,
 block_size = NULL
 ))
 cat("Done\n")

 \# Permuted block randomization (multiple block sizes)
 for(block_size in BLOCK_SIZES) {
 cat("	- Block size ", block_size, "... ", sep = "")
 blocked_results <- map_dfr(1:N_TRIALS, ~simulate_blocked_trial(
 cohort_data = cohort_df,
 cohort_name = cohort_name,
 trial_id = paste0(cohort_name, "_n", n, "_block", block_size, "_", .x),
 n_per_arm = n,
 threshold_mean_cfs = threshold,
 block_size = block_size
 ))
 cat("Done\n")

 all_results <- bind_rows(all_results, blocked_results)
 }

 all_results <- bind_rows(all_results, simple_results)
 }

 cat("\n")
}

\# ============================================================================
\# SUMMARIZE RESULTS
\# ============================================================================

cat("Calculating summary statistics...\n\n")

summary_by_method <- all_results %>%
 group_by(cohort, n_per_arm, randomization_method, block_size) %>%
 summarise(
 n_trials = n(),
 n_paradoxical = sum(paradoxical),
 pct_paradoxical = mean(paradoxical) * 100,
 n_distributionally_imbalanced = sum(distributionally_imbalanced),
 pct_distributionally_imbalanced = mean(distributionally_imbalanced) * 100,
 .groups = "drop"
 ) %>%
 rowwise() %>%
 mutate(
 ci_paradoxical = list(calc_proportion_ci(n_paradoxical, n_trials)),
 paradoxical_lower = ci_paradoxical$lower,
 paradoxical_upper = ci_paradoxical$upper
 ) %>%
 ungroup() %>%
 select(-ci_paradoxical)

\# ============================================================================
\# COMPARISON: SIMPLE vs BLOCKED
\# ============================================================================

cat("Comparing simple vs blocked randomization...\n\n")

\# Calculate reduction in paradoxical trials from blocking
comparison <- summary_by_method %>%
 select(cohort, n_per_arm, randomization_method, pct_paradoxical) %>%
 pivot_wider(
 names_from = randomization_method,
 values_from = pct_paradoxical
 ) %>%
 mutate(
 reduction_block4 = `Simple 1:1` - `Block size 4`,
 reduction_block6 = `Simple 1:1` - `Block size 6`,
 reduction_block8 = `Simple 1:1` - `Block size 8`,
 pct_reduction_block4 = (reduction_block4 / `Simple 1:1`) * 100,
 pct_reduction_block6 = (reduction_block6 / `Simple 1:1`) * 100,
 pct_reduction_block8 = (reduction_block8 / `Simple 1:1`) * 100
 )

cat("COMPARISON TABLE:\n")
cat("Absolute reduction in paradoxical trial prevalence (%)\n\n")
print(comparison %>%
 select(cohort, n_per_arm, `Simple 1:1`,
 reduction_block4, reduction_block6, reduction_block8) %>%
 mutate(across(where(is.numeric), ~round(.x, 1))))

cat("\n\nRelative reduction (%)\n\n")
print(comparison %>%
 select(cohort, n_per_arm,
 pct_reduction_block4, pct_reduction_block6, pct_reduction_block8) %>%
 mutate(across(where(is.numeric), ~round(.x, 1))))

\# ============================================================================
\# KEY FINDINGS
\# ============================================================================

cat("\n══════════════════════════════════════════════════════════════════\n")
cat("KEY FINDINGS\n")
cat("══════════════════════════════════════════════════════════════════\n\n")

\# Overall effectiveness of blocking
overall_comparison <- summary_by_method %>%
 group_by(randomization_method) %>%
 summarise(
 mean_paradoxical = mean(pct_paradoxical),
 .groups = "drop"
 )

cat("Overall mean paradoxical trial prevalence across all cohorts and sample sizes:\n\n")
print(overall_comparison %>% mutate(mean_paradoxical = round(mean_paradoxical, 1)))

\# Effect at specific sample sizes
cat("\n\nAt sample size n=100 per arm:\n")
print(summary_by_method %>%
 filter(n_per_arm == 100) %>%
 select(cohort, randomization_method, pct_paradoxical) %>%
 mutate(pct_paradoxical = round(pct_paradoxical, 1)))

cat("\n\nAt sample size n=250 per arm:\n")
print(summary_by_method %>%
 filter(n_per_arm == 250) %>%
 select(cohort, randomization_method, pct_paradoxical) %>%
 mutate(pct_paradoxical = round(pct_paradoxical, 1)))

\# ============================================================================
\# SAVE RESULTS
\# ============================================================================

write.csv(all_results, "block_randomization_all_trials.csv", row.names = FALSE)
write.csv(summary_by_method, "block_randomization_summary.csv", row.names = FALSE)
write.csv(comparison, "block_randomization_comparison.csv", row.names = FALSE)

cat("\n✓ Saved: block_randomization_all_trials.csv\n")
cat("✓ Saved: block_randomization_summary.csv\n")
cat("✓ Saved: block_randomization_comparison.csv\n\n")

cat("══════════════════════════════════════════════════════════════════\n\n")

### Output Files

The script generates three CSV files:

1. **block_randomization_all_trials.csv** - Individual trial results for each simulation
2. **block_randomization_summary.csv** - Summary statistics by cohort, sample size, and randomization method
3. **block_randomization_comparison.csv** - Direct comparison showing reduction in paradoxical trials from blocking

## STROBE checklist

STROBE Statement—checklist of items that should be included in reports of observational studies

|  | Item No | Recommendation | Page  No |
| --- | --- | --- | --- |
| **Title and abstract** | 1 | (*a*) Indicate the study’s design with a commonly used term in the title or the abstract | 1 |
|  |  | (*b*) Provide in the abstract an informative and balanced summary of what was done and what was found | 2 |
| Introduction | | | |
| Background/rationale | 2 | Explain the scientific background and rationale for the investigation being reported | 3-4 |
| Objectives | 3 | State specific objectives, including any prespecified hypotheses | 4 |
| Methods | | | |
| Study design | 4 | Present key elements of study design early in the paper | 4-6 |
| Setting | 5 | Describe the setting, locations, and relevant dates, including periods of recruitment, exposure, follow-up, and data collection | 4 |
| Participants | 6 | (*a*) *Cohort study*—Give the eligibility criteria, and the sources and methods of selection of participants. Describe methods of follow-up  *Case-control study*—Give the eligibility criteria, and the sources and methods of case ascertainment and control selection. Give the rationale for the choice of cases and controls  *Cross-sectional study*—Give the eligibility criteria, and the sources and methods of selection of participants | 4-8 |
|  |  | (*b*) *Cohort study*—For matched studies, give matching criteria and number of exposed and unexposed  *Case-control study*—For matched studies, give matching criteria and the number of controls per case |  |
| Variables | 7 | Clearly define all outcomes, exposures, predictors, potential confounders, and effect modifiers. Give diagnostic criteria, if applicable | 4-8 |
| Data sources/ measurement | 8* | For each variable of interest, give sources of data and details of methods of assessment (measurement). Describe comparability of assessment methods if there is more than one group | 4-8 |
| Bias | 9 | Describe any efforts to address potential sources of bias | 5-6 |
| Study size | 10 | Explain how the study size was arrived at | NA |
| Quantitative variables | 11 | Explain how quantitative variables were handled in the analyses. If applicable, describe which groupings were chosen and why | 5-6 |
| Statistical methods | 12 | (*a*) Describe all statistical methods, including those used to control for confounding | 8-9 |
|  |  | (*b*) Describe any methods used to examine subgroups and interactions | 6-8 |
|  |  | (*c*) Explain how missing data were addressed | Fig 1 |
|  |  | (*d*) *Cohort study*—If applicable, explain how loss to follow-up was addressed  *Case-control study*—If applicable, explain how matching of cases and controls was addressed  *Cross-sectional study*—If applicable, describe analytical methods taking account of sampling strategy | NA |
|  |  | (*e*) Describe any sensitivity analyses |  |

Continued on next page

| Results | | | |
| --- | --- | --- | --- |
| Participants | 13* | (a) Report numbers of individuals at each stage of study—eg numbers potentially eligible, examined for eligibility, confirmed eligible, included in the study, completing follow-up, and analysed | Fig 1 |
|  |  | (b) Give reasons for non-participation at each stage | Fig 1 |
|  |  | (c) Consider use of a flow diagram | Fig 1 |
| Descriptive data | 14* | (a) Give characteristics of study participants (eg demographic, clinical, social) and information on exposures and potential confounders | Table 1 |
|  |  | (b) Indicate number of participants with missing data for each variable of interest | Table 1 |
|  |  | (c) *Cohort study*—Summarise follow-up time (eg, average and total amount) | Table 1 |
| Outcome data | 15* | *Cohort study*—Report numbers of outcome events or summary measures over time | Table 1 |
|  |  | *Case-control study—*Report numbers in each exposure category, or summary measures of exposure |  |
|  |  | *Cross-sectional study—*Report numbers of outcome events or summary measures |  |
| Main results | 16 | (*a*) Give unadjusted estimates and, if applicable, confounder-adjusted estimates and their precision (eg, 95% confidence interval). Make clear which confounders were adjusted for and why they were included | 9-11 |
|  |  | (*b*) Report category boundaries when continuous variables were categorized | NA |
|  |  | (*c*) If relevant, consider translating estimates of relative risk into absolute risk for a meaningful time period | NA |
| Other analyses | 17 | Report other analyses done—eg analyses of subgroups and interactions, and sensitivity analyses | Additional file 1 |
| Discussion | | | |
| Key results | 18 | Summarise key results with reference to study objectives | 11 |
| Limitations | 19 | Discuss limitations of the study, taking into account sources of potential bias or imprecision. Discuss both direction and magnitude of any potential bias | 14-15 |
| Interpretation | 20 | Give a cautious overall interpretation of results considering objectives, limitations, multiplicity of analyses, results from similar studies, and other relevant evidence | 15 |
| Generalisability | 21 | Discuss the generalisability (external validity) of the study results | 14-15 |
| Other information | | | |
| Funding | 22 | Give the source of funding and the role of the funders for the present study and, if applicable, for the original study on which the present article is based | 16 |

*Give information separately for cases and controls in case-control studies and, if applicable, for exposed and unexposed groups in cohort and cross-sectional studies.

**Note:** An Explanation and Elaboration article discusses each checklist item and gives methodological background and published examples of transparent reporting. The STROBE checklist is best used in conjunction with this article (freely available on the Web sites of PLoS Medicine at http://www.plosmedicine.org/, Annals of Internal Medicine at http://www.annals.org/, and Epidemiology at http://www.epidem.com/). Information on the STROBE Initiative is available at www.strobe-statement.org.
